# Neutral fitness outcomes contradict inferences of sexual ‘coercion’ derived from male’s damaging mating tactic in a widow spider

**DOI:** 10.1038/s41598-017-17524-6

**Published:** 2017-12-11

**Authors:** Luciana Baruffaldi, Maydianne C. B. Andrade

**Affiliations:** Departments of Biological Sciences and Ecology & Evolutionary Biology, University of Toronto Scarborough, Toronto, ON M1C 1A4 Canada

## Abstract

Sexual conflict over mating frequency has driven the evolution of morphological and behavioural traits across taxa. Interactions may be termed ‘coercive’ and assumed to arise from conflict when male mating behaviours cause physical injury to females and females appear to resist injurious matings.However, coercion per se occurs only if the behaviour reduces female fitness; and such outcomes are rarely measured. Here we show that a damaging mating tactic, apparently adaptive for males, is not coercive for females. Adult male *Latrodectus* spiders mate with immature females after tearing the exoskeleton covering the female’s recently-developed reproductive tract, which can cause haemolymph bleeding. We show that, relative to pairings with adult females, males use reduced courtship displays when approaching immature females, which in some cases respond with elevated deterrent behavioural responses. Nevertheless, we found no reproductive cost for immature-mated females in terms of longevity, fertility or fecundity. Moreover, most immature-mated females did not produce sex pheromones as adults, so did not seek additional matings. Thus, despite the appearance of conflict there is no evidence that immature-mating is coercive. These results show it is critical to measure fitness outcomes, in addition to behavioural responses, to test for coercion.

## Introduction

Sexual selection on males has driven the evolution of a wide range of courtship behaviors that may persuade females to mate^1^. In some species, intense sexual conflict over mating frequency has instead produced male structural adaptations and behaviours for mating while bypassing persuasive courtship and other forms of mating effort^2,3^. Such matings are considered ‘coercive’ if they decrease female fitness relative to forgoing mating^2–4^, but may occur nonetheless if the direct costs of resistance by females outweigh the fitness costs of mating^2–4^. Although males of some species show almost exclusively coercive tactics, in other species, these tactics may be adopted only in certain contexts or only by certain males^2,5^. Evidence for coercive mating has accumulated, primarily through studies in insects (e.g.^6^), with additional proposed examples including birds (e.g.^7^), fish (e.g.^8^) and snakes (e.g.^9^). However, coercion is expected to coincide with intense sexual conflict^2^, and a recent review suggests the estimated prevalence of conflict is decreasing as the number of taxa in which it is studied increases^10^. It is problematic that in many taxa, data on fitness effects of mating are challenging to acquire, and in these, sexual conflict and coercion is inferred indirectly, often from female behaviour (e.g., primates^11^; fish^8^).

Coercion may be inferred when females show distinct ‘deterrent’ behaviours in response to male mating attempts; females may shake their bodies, move rapidly, or even fight with males before or during mating^4,6,12^. Deterrent behaviours may cause increased energy expenditure or predation risk^6,12^ for females, but may evolve if they allow avoidance of costly matings with coercive males. However, these behaviours may also be a mechanism of female choice if persistent or physically powerful males are superior mates^4,12,13^. Thus, although females are predicted to resist coercive mating, it is not possible to distinguish the function of female deterrent behaviours only by observation. By extension, it not possible to determine whether matings are coercive based on female responses. The critical question is whether or not females suffer net fitness deficits when they mate with males that use coercive tactics^13,14^.

**Figure 1:**
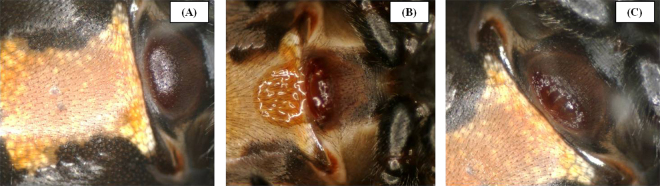
The external genitalia of *L*. *hasselti* females, located on the ventral surface of the abdomen just anterior to the upper margin of the ‘hourglass’ marking, and epigastric furrow. In all images, the posterior is left, anterior is right. (**A**) Protuberant genital region of an immature female two days before molt to adulthood; no external openings are present. (**B**) Genital region of immature female after mating with an adult male with haemolymph visible leaking from the region that was opened by the male. (**C**) Epigynum (mature external genitalia) of an adult unmated female with genital openings uncovered following the final moult.

Here, we ask whether a recently-described, damaging mating tactic of male spiders^15^ may be coercive, and whether this is consistent with observations of male and female behaviour, as well as female fitness outcomes. In some *Latrodectus* spiders (*L*. *hasselti* and *L*. geometricus^15^) males use an ‘immature-mating’ tactic, in which they mate with females in their final juvenile instar (immature females) by tearing the exoskeleton covering the female’s fully-developed, but otherwise concealed genitalia^15^, sometimes causing haemolymph ‘bleeding’ (LB, pers obs, Fig. 1). Immature-mated females store sperm through their final moult, then produce fertilized eggs. In the Australian redback spider *Latrodectus hasselti*, at least 1/3 of immature females are mated in this way in the field^15^. This behaviour can be staged in the laboratory^15^, where we were able to examine male and female behaviour and fitness consequences of mating in detail.

In addition to the damage caused to immature-mated females (Fig. 1), immature mating may be inferred to be coercive because courtship is absent and female choice behaviour appears to be constrained. In previous work, immature-mating (IM) males were reported not to court, but were rarely cannibalized by females^15^. In contrast, when *L*. *hasselti* males mate with adult females (AM), prolonged vibratory courtship is typical, and males that attempt copulation early are killed by females before mating is complete (premature cannibalism^16^). However, there has been no detailed analysis of immature-mating behaviours to date, so it is unclear how males change their behaviour when approaching immature females, or whether females engage in deterrent behaviours (see^16^). Moreover, it is not clear whether immature mating affects future mate attraction, nor whether the damage caused to immature females (Fig. 1) decreases longevity (an important fitness component).

Here we examined whether the components of courtship employed by males approaching immature females are reduced relative to males courting adults, and whether immature females engage in behaviours that may slow male progress towards mating (although this in itself does not demonstrate coercion^13^). Second, we investigated effects of immature mating on female sex pheromone production following mating. As found in other invertebrates, unmated adult females produce sex pheromones that attract mates and trigger courtship^17,18^, but mated females cease sex pheromone production after mating as adults, and hence stop attracting males^16^. If immature mating is coercive, we predicted immature-mated females would signal for additional males as adults (i.e., after their final moult). Third, we test a critical prediction of the coercive mating hypothesis; that immature-mated females will have lower fitness (measured as fecundity, fertility, and post-mating survivorship) than females mated as adults.

## Results

We randomly assigned laboratory-reared *L*. *hasselti* females to one of 3 experimental groups that differed in mating treatment: (1) Immature females mated with adult males during their final immature instar (IM); (2) Adult females mated with adult males after their final adult moult (AM); and (3) Unmated adult females were never exposed to males and never mated (V). Mating trials of IM and AM females were recorded on digital video. We compared behaviours from mating trials with complete video records, and which were known to involve successful copulation and sperm transfer because females produced egg sacs with viable offspring (Immature-mated, IM, n = 18; Adult-mated, AM, n = 17). To be conservative, we excluded females that appeared to mate but did not produce fertile eggs because it was possible that such females were not sufficiently developed at the time we paired them with males, and thus were incapable of storing sperm (see^15^).

The time from the start of trials until the first copulation was similar for IM and AM trials^15^, but male and female behaviours were very different (Table 1). Latency to the first copulation for AM females was mainly taken up with male courtship. In contrast, for IM females, this latency was mainly because males’ initial approach to females was slower, with some males taking longer to mount immature females (Table 1). Female-initiated responses to male mounting and mating attempts (deterrent behaviours) were observed in immature and adult females (Table 1). Deterrent behaviours included females rapidly raising and lowering their legs (usually the anterior and posterior-most legs), and hitting the male or the web near the male (‘strikes’, see^19^), and females moving their posterior legs and body while the male was mounted, rather than remaining quiescent. Males responded to these deterrent behaviours by (1) moving away from the female (if on the web), or (2) quitting the female’s abdomen and returning to the web before making another mounting attempt or (3) remaining on the female’s abdomen but moving away from contact with the female’s legs. There was broad variation in the frequency of the deterrent behaviour of females (Table 1). IM females showed more variation in their response to approaching males than did AM females, with some IM females exhibiting extremely high frequencies of deterrent behaviours. Our analysis suggests, overall, that IM females have elevated leg and body movements in response to male mounting attempts prior to the first copulation relative to AM females (Table 1).

**Table 1:** Description and comparison (mean ± s.d.) of male and female-initiated behaviours and mating outcomes scored for pairings between adult males and immature or adult females.

Variable^*^	Description	Immature mated	Adult mated	Statistics
**Mount Latency (min)**	**Time from initiation of trial until males first climb onto the female’s ventral abdomen (close to the female’s genitalia)**	**86**.**55 ± 112**.**74 (18)**	**24**.**31 ± 38**.**35 (16)**	**GLMS Wald X ** ^**2**^ ** = 11**.**32**, **p = 0**.**001**
**Number of Mounts 1**	**Number of times males move onto female’s ventral abdomen prior to the first copulation**.	**5**.**72 ± 10**.**64 (18)**	**21**.**53 ± 8**.**22 (15)**	**GLMS Wald X ** ^**2**^ ** = 137**.**16**, **p < 0**.**001**
**Number of occurrences of deterrent behaviours**	**Number of times females engage in strikes**, **leg or body movement prior to the first copulation**	**17 ± 25**.**84(18)**	**9**.**19 ± 14**.**28(16)**	**GLMS Wald X ** ^**2**^ ** = 37**.**60**, **p < 0**.**001**
Copulation 1 Latency (min)	Time from start of trial to start of first copulation.	118.2 ± 101.97 (15)	129.69 ± 61.51 (16)	GLMS Wald **X** ^2^ = 0.141, p = 0.707
Copulation 1 Duration (min)	Total duration of the first copulation.	19.93 ± 5.09 (15)	16.36 ± 6.44 (14)	GLMS Wald **X** ^2^ = 2.936, p = 0.087
**Number of Mounts 2**	**Number of mounts between the first and second copulation**.	**0**.**13 ± 0**.**35 (15)**	**2**.**42 ± 2**.**15 (12)**	**GLMS Wald X ** ^**2**^ ** = 15**.**705**, **p < 0**.**001**
**Copulation 2 Latency (min)**	**Time from end of first copulation to start of second copulation**.	**12**.**5 ± 16**.**14 (14)**	**27**.**91 ± 9**.**36 (11)**	**GLMS Wald X ** ^**2**^ ** = 5**.**275**, **p = 0**.**022**
Copulation 2 Duration (min)	Total duration of second copulation.	24.43 ± 7.26 (14)	19.4 ± 7.53 (10)	GLM F = 2.714, p = 0.114

^*^Variables in bold differ significantly between the two treatments.

All males approaching AM females (n = 17) initially courted on the web, continuously moving their abdomen and adding their own silk to the adult female’s web. All males then alternated between courtship on the web and courtship while mounted on the AM female’s body, repeating the sequence 21 times on average before the first copulation (Table 1, also see^16^). In contrast, 50% of IM females (n = 18) were approached without any vibratory courtship on the web (compared to AM females: χ^2^ = 11.44, p < 0.001), and when courtship occurred on the web, it was mainly brief abdominal vibrations. After mounting IM females, males typically remained there and rarely returned to the web, with only 28% of males alternating between courtship on the web and on the female’s body (less common than for males that mounted AM females: χ^2^ = 18.71, p < 0.001; Table 1).

Despite the absence of pre and post-mounting courtship by males in IM compared to AM trials (Table 1), males mated to IM females fared better on most indicators of male fitness^15^. Overall, males mated to IM females were more likely to copulate twice and thus inseminate the female’s paired perm storage organs (spermathecae; 88% of IM trials compared to 75% of AM trials, see^15^), which can increase paternity^16^. In normal AM matings, *L*. *hasselti* males twist their body over the female’s mouthparts during copulation (‘somersault’) and are frequently cannibalized, limiting other mating opportunities^15^. Here, 94% of males somersaulted with AM females, whereas only 12% of males somersaulted in first copulations with IM females (χ_1_^2^ = 22.181, p < 0.001). Moreover, AM females killed 25% of males during their first copulation (premature cannibalism^19^) and 17% during the second copulation (n = 12). In contrast, IM females never killed males during the first nor second copulation.

Whereas 10 of the 12 surviving AM males (83%) returned to the web after the first copulation and resumed courtship prior to attempting a second copulation, only 13% (n = 15) of IM males dismounted females prior to the second copulation (χ_1_^2^ = 13.230, p < 0.001). As a result, IM males that mated twice did so much more rapidly than AM males that mated twice (Table 1).

### Sex pheromone bioassay

**Figure 2:**
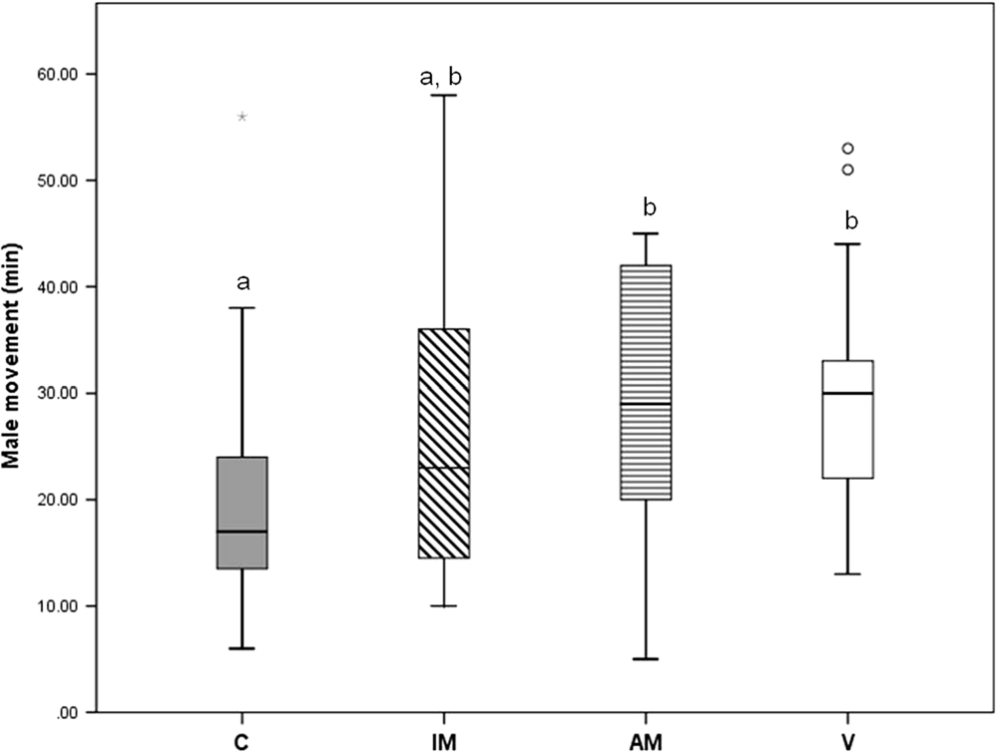
Comparison (median, interquartile range) of male searching behaviour on silk extracts from unmated females (V) (white box, n = 23), adult mated females (AM) (horizontal striped box, n = 16), immature mated females (IM) (diagonal striped box, n = 23), and control (methanol, C) (gray box, n = 23). Different letters above box plots show significantly different outcomes (p < 0.05, post-hoc test).

Within two weeks after their final moult, silk was harvested from all females and contact pheromones extracted^20^. Extracts were used in a standard bioassay of male activity which examines male mate-searching movements on extracts containing pheromones, or solvent alone [see^20–22^]. Male mate-searching responses were graded across treatments (Kruskal Wallis test = 8.727, df = 3, p = 0.033, Fig. 2), with the most intense response to extracts of unmated adult females (V), intermediate responses to AM females, and the least intense response to IM adult females (not different from controls, Kruskal Wallis Pair-wise test = −9.935, p = 0.172, Fig. 2). Activity in the IM and AM groups was similar (Kruskal Wallis Pair-wise test = 8.541, p = 0.287), with broad, overlapping variation in male responses (Fig. 2).

### Fitness effects for females

**Figure 3:**
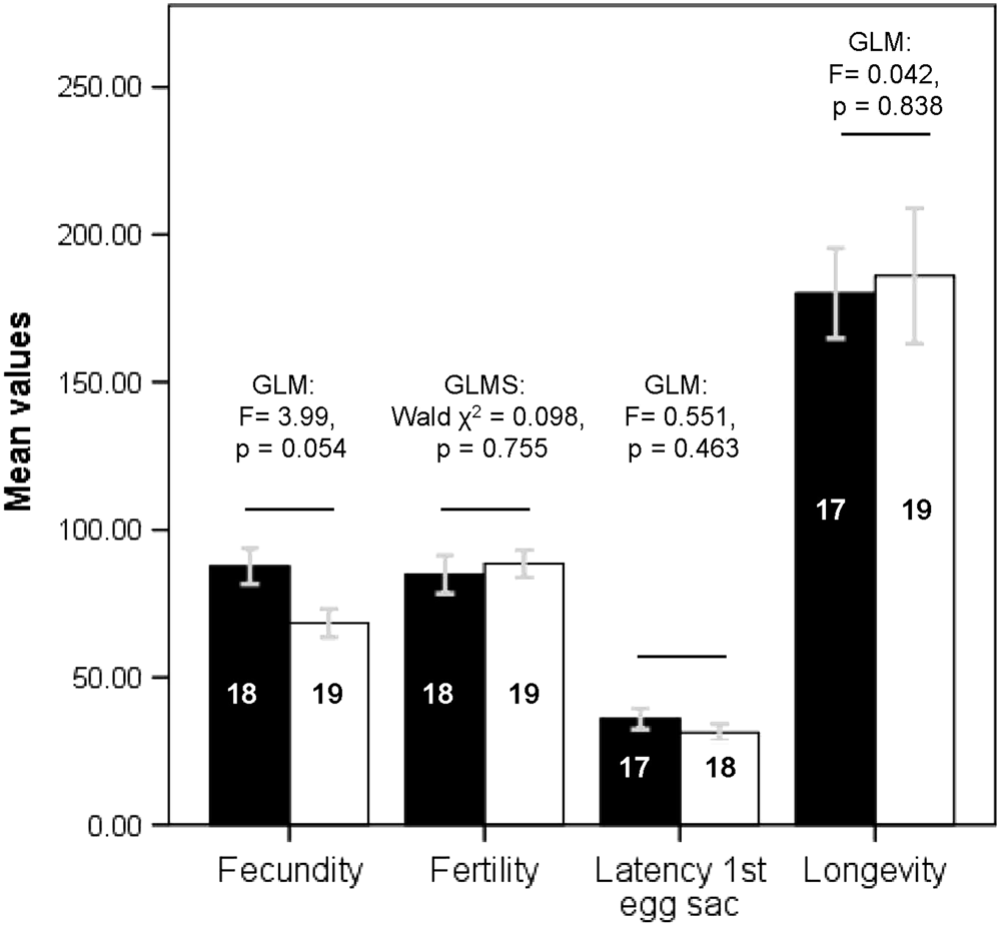
Mean (±s.e.) of female fecundity (number of eggs produced), fertility (% of eggs that hatch, note that the maximum value is 100), latency to produce the first egg sac (days) and longevity (days) for immature mated females (IM, white bars) and adult mated females (AM, black bars). Statistics above each pair of bars show the results of comparisons of IM and AM females within each category (GLM = general linear model; GLMS = generalized linear model).

All females were kept on a common diet, and their longevity was recorded. For AM and IM females, the first egg sac produced was collected and fecundity and fertility estimated from this egg sac. Mated *L*. *hasselti* females are not sperm limited, and produce egg sacs throughout their post-mating lives at regular intervals that depend on body condition and diet^23^. However, the number of spiderlings per egg sac depends on body size and remains constant until females begin to senesce^23^. Thus examining the first egg sac and female longevity together provide good insight into reproductive fitness. IM females were similar to AM females in latency to deposit their first egg sac, fecundity and fertility (Fig. 3, and see^15^). Critically, despite exoskeletal tearing (Fig. 1), IM females had similar longevity to AM females (Fig. 3).

## Discussion

*Latrodectus hasselti* males approach and mount females before females are mature, with minimal courtship (^15^, Table 1), and breach the female’s exoskeleton in order to mate (^15^, Fig. 1). These tactics are met with deterrent behaviours by females (Table 1), but nonetheless, we have found that immature-mating does not lead to significant fitness deficits for females and thus, by definition^2^ is not coercive. Males that mated with immatures showed little evidence of the continuous, web-based courtship seen when males approach adults^19–24^. Rather, males approached immature females without much signalling (also see^15^), mounted, then attempted to remain mounted until two copulations were achieved. Nevertheless, compared to males paired with adults, males that successfully mated with immatures were more likely to achieve two copulations^15^, which predicts higher paternity if females are polyandrous^16^. This was mediated by a significant difference in female cannibalism of males after the first copulation (‘premature cannibalism; 0% IM; 25% AM). Moreover, whereas males show strong responses to pheromones produced by unmated adult females, it appears that IM females produce relatively little sex pheromone as adults (Fig. 2). Finally, being mated as immatures was not costly for females neither in terms of their reproductive output^15^ nor survival (Fig. 3). Thus, we conclude that immature mating is not coercive, and other hypotheses for female ‘deterrent’ behaviours (observed in immature and adult females), such as mate choice, should be explored^25^.

We expected that if immature mating (IM) was coercive, IM females would seek additional mating partners as adults, and so exhibit similar patterns of pheromone production as unmated (V) females^21^. However, responses to extracts from the silk of IM females were variable, overlapping both the controls and the other treatment groups. This result was made more challenging to interpret because, contrary to previous work^21^, we found some AM females produce pheromones that trigger mate-searching by males (Fig. 2). The broad range of male responses to AM extracts overlapped responses to V and IM females. Thus, although on average IM females show a reduction in pheromone-mediated attractiveness after mating, (some of them) may still be attractive to males (Fig. 2). In *Drosophila melanogaster*, females that were coerced to mate apparently continued to be attractive to males after copulation, presumably through pheromone production, while those able to choose their first mate ceased to be attractive^26^. Our results are not as clear cut, and future work will examine the sources of variation among females in pheromone production, and potential links to phenotype of the first mate, polyandry, and age^27^.

Overall, we conclude that immature mating is neutral or beneficial to female fitness in *L*. *hasselti*. Possible benefits of immature-mating for females include reproducing without waiting to attract a male after moulting. Fertility assurance is important since approximately 17% of *L*. *hasselti* females remain unmated in nature, a risk that could significantly affect selection on female behaviour^16,28^. This risk is compounded by the fact that unmated adult *L*. *hasselti* females suffer decreased longevity relative to mated females^29^, increasing the cost associated with delays to mating.

Another possible benefit may arise if males that are able to achieve matings with immatures confer superior traits on offspring. The ability to locate and identify immature females in the narrow time window when this type of mating is possible in nature^15^ may require superior sensory or locomotory traits. Similarly, successfully opening the female’s genitalia without causing lasting damage requires coordination between the sensory and motor organs. Both of these abilities could be indicators of a high quality mate^14,25^. In our study, as in others, it is difficult to distinguish between female efforts to avoid unwanted copulations and resistance to copulatory attempts functioning as female choice^14,25^. Although we were unable to assess it here, deterrent behaviours may ensure that only persistent, vigorous males are able to mate; similar to other typical mechanisms to evaluate male quality^14,25^. If these traits are heritable, then IM females may benefit if their male offspring are also more likely to achieve immature matings (e.g^30^.). The difficulty with testing these hypotheses lies partly in the interpretation of failed matings, since it can be challenging (for researchers) to identify mating-capable immature females *a priori*.

These results suggest the importance of simultaneous analysis of inter-sexual behavioural interactions and fitness correlates, and interpretation in terms of natural history. Although this is emphasized in theory, it is not uncommon to see behavioural functions inferred based on the apparently antagonistic nature of the interactions^13^. Broadening studies to include a range of taxa on which it is possible to estimate female fitness may be critical to progress in understanding the evolutionary origin and maintenance of coercion.

## Methods

*Latrodectus hasselti* (Thorell 1870) spiders from an outbred laboratory population (started with mated females collected in Sydney, Australia) were reared following standard methods^22^ and their development tracked. In adult females, the raised, sclerotized external genitalia (epigynum, Fig. 1) includes separate openings leading to bilaterally paired copulatory ducts that empty into two separate sperm storage organs (spermathecae). We identified immature females capable of mating when their developing genital structures caused a raised bump to form in the overlying exoskeleton of the ventral abdomen (Fig. 1a)^15^. We randomly assigned such females to one of 3 experimental groups: (1) Immature-mated (IM) females were paired with adult males during their immature instar, 2 to 4 days before their final moult to adulthood (precise interval determined post-hoc), (2) Adult-mated females (AM) females were paired with males 3 to 10 days after their final adult moult, and (3) Unmated females (V) remained isolated from males so they did not mate. Mating trials and pheromone bioassays occurred during the dark phase, were recorded using digital macro-videography (Panasonic WV BP330 cameras with Navitar 7000 macro-zoom lenses, see) under red light [details in^19,22^].

### Mating trials

IM and AM females were placed in mating arenas to construct webs on metal frames (11 × 8 × 8 cm). Trials commenced when males were placed on a web and ended after mating occurred or after 8 hours^19^. We quantified behaviours that reflected mating progress from male approach through copulation^19,24^ (Table 1). Redback males need to copulate twice to inseminate both sperm storage organs (which increases the likelihood of sperm precedence^31^), but females need copulate only once to fertilize all of their eggs^23^. Males may court for several hours before attempting the first copulation with adult females^19^, they then dismount and court again before mounting the second time and attempting a second copulation^24^. We quantified male courtship, mounting and copulation behaviours and latencies. During courtship, receptive adult females are generally quiescent^19,24^. In contrast, some adult females strike at approaching males with their forelegs, and are less likely to mate than are quiescent females^19,32^. We recorded foreleg strikes and other forms of female activity during male approach or mounting. Sample sizes vary (Table 1) because it was sometimes not possible to extract certain types of data from videos.

### Pheromone extraction and bioassay

Courtship or mate-searching behaviour is triggered when males contact a web-borne pheromone present on the silk of unmated females, but reportedly absent from the silk created after females copulate as adults^21^. Within two weeks after the adult moult day, females from all treatment groups were placed in clean silk-collection arenas (details in^22^). After four days, silk was harvested, and submerged in 0.15 ml of methanol (HPLC, 99.9%, Fisher Chemicals) for 24 h to extract sex pheromones^22^.

The presence and attractiveness of the sex pheromones produced by experimental females was estimated by the duration of total male movement in response to silk extracts during 60 minute trials [see^21,22^]. Virgin males were placed on filter paper treated with the 0.15 ml of silk extract (silk + methanol) or methanol alone (control, C) inside clean glass petri plates (90 mm). Each spider was used as a silk source (females) or assayed (males) once. Pheromone assays included extracts from virgin females and only those mated females (IM and AM) that produced viable egg sacs (and so were confirmed to have mated successfully).

### Fitness effects of mating for females

Mated IM and AM females were fed adult crickets (*Gryllodes sigitalis*) once each week until their natural death. The first egg sac was removed, then frozen following spiderling emergence (reproductive output per egg sac does not vary significantly across the first 15 sacs produced in the lab^23^). Female fecundity (total eggs produced = spiderlings + unhatched eggs), fertility (100* hatched spiderlings divided by fecundity), latency to produce the first egg sac, and post-mating longevity (number of days since adult moult for IM females, and since mating for AM females) was compared between mating treatments, in models that included female mass as a covariate.

### Statistical analysis

For mating trials, General Linear Models (GLM) were used to analyze normally distributed data, Generalized Linear Models (GLMS) was used for analyzing durations (Gamma distribution with log link), and occurrence data (Poisson distribution with log linear link). χ^2^ tests were used for behavioral frequencies (IBM SPSS Version 22). Pheromone response data were non-normal and assessed using the Kruskal Wallis test.

### Data accessibility

Raw data are available from the Dryad Digital Repository: doi:10.5061/dryad.cr785.
